# Unusual ectasia following corneal cross-linking in a patient with keratoconus

**DOI:** 10.22336/rjo.2025.50

**Published:** 2025

**Authors:** Lorena Azorin-Perez, Ana Hervas-Ontiveros, Javier Cañas-Costa, Marta Gema Solaz-Ruiz, Pablo Morales-Lopez, Enrique España-Gregori

**Affiliations:** 1 La Fe University and Polytechnic Hospital, Valencia, Spain; 2 Clinical and Surgical Institute of Ophthalmology, Valencia, Spain

**Keywords:** ectasia, corneal ectasia, cross-linking, keratoconus, anterior segment, CXL = cross-linking, epi-off = epithelium-off, UVA = ultraviolet A, OD = Right eye, OS = Left eye, BCVA = best-corrected visual acuity

## Abstract

**Objective:**

Description of an unusual ectasia following corneal cross-linking (CXL) in a patient with keratoconus.

**Methods:**

Clinical case report and review of the literature.

**Results:**

We present a case of an unusual ectasia after corneal CXL in a patient with keratoconus.

**Discussion:**

This case reports the first known occurrence of corneal ectasia following epi-off CXL, a procedure typically used to halt the progression of ectatic disorders such as keratoconus. Although the standard protocol was followed, subtle technical variations or postoperative healing factors could have contributed to the outcome.

**Conclusions:**

This case underscores the need for ongoing vigilance in patient selection and follow-up in the context of CXL. The rare development of corneal ectasia post-CXL highlights a gap in our understanding of corneal biomechanics and the potential limitations of current diagnostic tools. More research is needed to refine patient screening methods and optimize treatment protocols to ensure the efficacy and safety of this widely used procedure.

## Introduction

CXL has revolutionized the treatment of keratoconus and other progressive corneal ectasias by enhancing corneal rigidity through the induction of cross-links between collagen fibers. One of the most widely accepted techniques for performing CXL is the epithelium-off (epi-off) approach, also known as the Dresden protocol, which has shown efficacy in halting ectatic progression in most cases. This procedure involves the removal of the corneal epithelium to allow better penetration of riboflavin into the stroma, followed by ultraviolet A (UVA) irradiation to initiate the cross-linking process [1,2].

Although CXL has been associated with a low risk of complications, adverse events such as infectious keratitis, corneal haze, and epithelial healing delays have been reported [3-5]. However, corneal ectasia following CXL has not been described in the literature, making this case particularly noteworthy [5].

This report presents the first known case of corneal ectasia developing after epi-off CXL, highlighting the importance of rigorous patient selection and thorough understanding of corneal biomechanics. Potential mechanisms for this complication were discussed, and modifications to current protocols that may reduce the risk of such outcomes in the future were suggested.

## Methods

We described a clinical case and a review of the literature.

## Results

A 45-year-old male was referred to our center from primary care in 2023 for follow-up of keratoconus. He was previously treated (7 years before) at a private clinic with intra-stromal rings in the right eye (OD) and cross-linking in the left eye (OS). The patient did not report any prior refractive surgeries or any ophthalmological problems.

The patient presented with progressive vision loss in the left eye, without other symptoms.

**Fig. 1:**
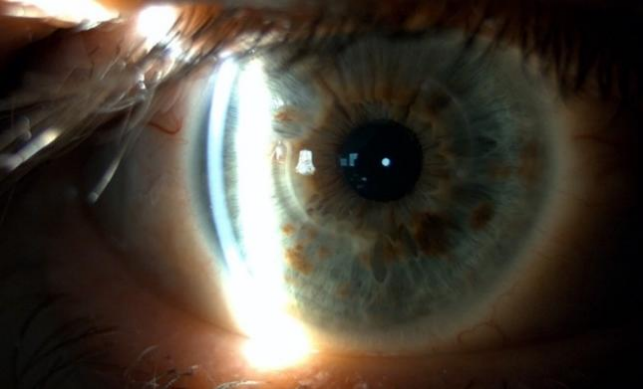
Slit lamp of OD. Well-positioned rings can be observed in the cornea

**Fig. 2:**
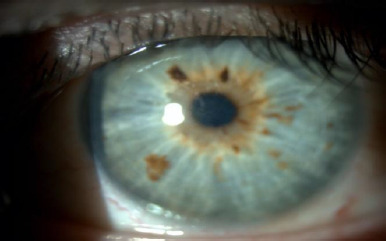
Slit lamp of OS. Central and endothelial oedema can be observed

**Fig. 3:**
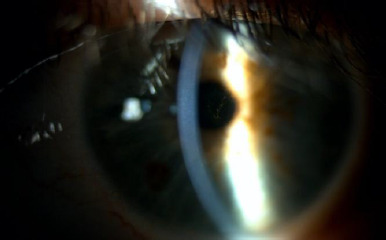
Slit lamp of OS. Diffuse pigment in the cornea

Upon ophthalmological examination, he had a best-corrected visual acuity (BCVA) of 0.1 LogMAR in the OD and 0.3 LogMAR in the OS. The refraction was -0.75 (-2.5 x 5º) in OD and +2 (-1.75 x 25º) in OS. In the slit lamp examination of the OD, two 90-degree well-positioned rings were observed without extrusion, and no other findings were noted (**Fig. 1**). In the OS eye, we found central endothelial and stromal oedema (**Fig. 2**) with diffuse pigment (**Fig. 3**).

**Fig. 4:**
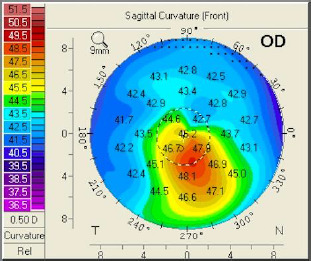
Topography of OD. A very mild inferior cone

**Fig. 5:**
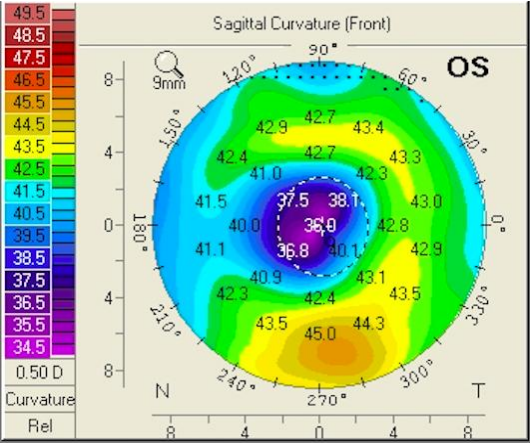
Topography of OS. Pattern of central ablation

Topographies were taken for future follow-up. In the topography of the right eye, we observed a very mild inferior cone (**Fig. 4**), while in the left eye, there was a pattern of central ablation (**Fig. 5**).

**Fig. 6:**
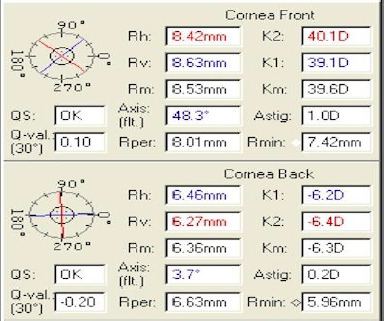
Main page analysis. Quality Score (QS) is correct. K-readings in the central 3 mm are low. Corneal thickness at the thinnest location is abnormal

Primary page analysis (**Fig. 6**) revealed that the quality of the capture (QS) was correct. The K-readings in the central 3 mm were low (40.1, 39.1), and the corneal astigmatism (algebraic sum of anterior and posterior astigmatism) was normal (1.2). Q-value at the 6 mm circle of the cornea front was also normal (0.10). Corneal thickness at the thinnest location was abnormal (460).

**Fig. 7:**
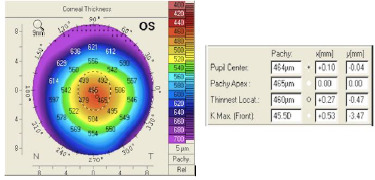
Corneal thickness map OS. The thinnest location is lower than 470 µm

**Fig. 8:**
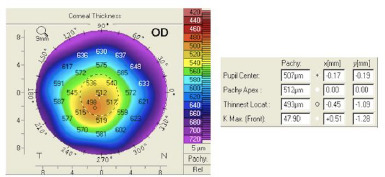
Corneal thickness map OD

Studying each map, we observed that the shape of the corneal thickness map (**Fig. 7**) was abnormal, with the thinnest location lower than 470 microns (µm), and the difference in thickness between the patchy apex and the thinnest location was a suspected value (5 µm). The thinnest location difference between both eyes was more than 30 µm as well (thinnest location in OD was 493 and in OS was 460 (**Fig. 8**). Furthermore, according to Rowsey’s rule of 2’s, the peripheral thickness should not be > 20 µm more than the central thickness, which was obviously absent in this case.

**Fig. 9:**
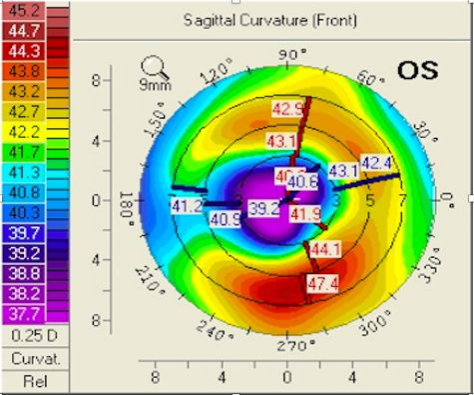
Sagittal curvature front map OS projecting the major axes

**Fig. 10:**
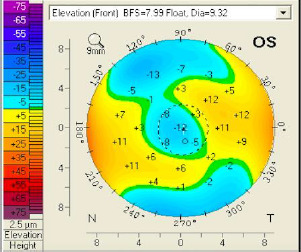
Elevation front map OS. A central flattening can be observed

**Fig. 11:**
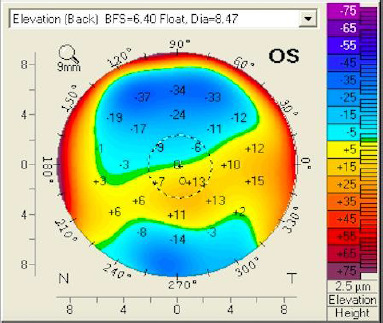
Elevation back map OS

The sagittal curvature front map (**Fig. 9**) showed abnormal values because there was a central flattening more pronounced at the inferior level. When projecting the major axes on that map, one could observe that the difference between the upper and lower points at the 6 mm circle was 4.5 dpt (normal values are 1.5 dpt or less). At the elevation front map, we could also observe a central flattening with a suspected value of -12 µm (**Fig. 10**). The elevation back map showed normal values (**Fig. 11**).

**Fig. 12:**
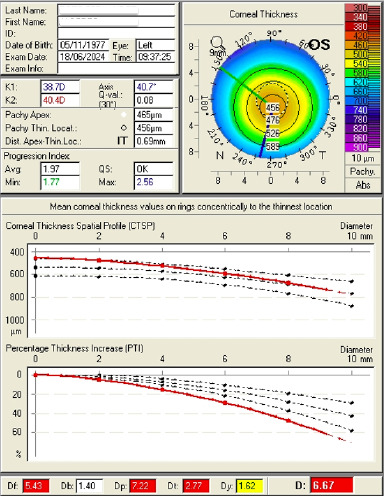
Keratoconus indices page OS

In the keratoconus indices page (**Fig. 12**), we could observe the abnormal values of the percentage thickness increase. The values deviated from normality the more peripheral they were.

An endothelial cell count was also performed, showing values within normal limits in the OD (cell density (CD) 2851) and an abnormality in the OS (CD 1796).

After the evaluation, the diagnosis of corneal ectasia following CXL at OS was performed, and treatment was initiated in the left eye with Sodium Chloride: 5% Sodium Hyaluronate: 0.15% (ODM5), three times a day, and dexamethasone 0.1% (Maxidex) twice a day. In the right eye, a contact lens was placed to improve the BCVA.

After four months of treatment, the patient was scheduled for a follow-up appointment. This time, during the ophthalmological examination, we found a BCVA of 0.0 LogMAR in OD and 0.1 LogMAR in OS. The slit lamp examination revealed a slight improvement in the oedema.

## Discussion

This case presents the first known report of corneal ectasia following epi-off CXL. Typically, CXL is performed to prevent or arrest the progression of ectatic corneal disorders such as keratoconus [2]. In this patient, the unexpected development of post-CXL ectasia raises essential questions about the underlying mechanisms and patient selection criteria for the procedure.

The most plausible explanation for the development of ectasia in this case may involve intrinsic biomechanical abnormalities in the cornea that were not detected preoperatively [6]. Although preoperative assessments, including corneal tomography and pachymetry, indicated that the patient was an appropriate candidate for CXL, these evaluations may not always reveal subtler, underlying biomechanical weaknesses. Studies have demonstrated that certain corneas with apparently normal preoperative parameters may still be predisposed to ectasia due to subclinical conditions or undiagnosed biomechanical fragility [5].

Additionally, variations in the application of the CXL protocol, including differences in UVA fluence, riboflavin saturation, or corneal hydration, could theoretically result in uneven cross-linking effects, potentially contributing to focal areas of biomechanical instability [7]. However, based on the information from the original center, the standard protocol was followed without deviation, suggesting that this was less likely to be the cause in this case.

Another potential factor to consider is the patient’s postoperative care. Although the patient followed the standard postoperative regimen, including the use of topical corticosteroids to control inflammation, the possibility of suboptimal healing or delayed epithelial recovery cannot be ruled out. The role of postoperative corneal biomechanics in influencing long-term outcomes following CXL has yet to be fully elucidated, and further research is needed to explore this area [8,9].

## Conclusion

In conclusion, this case underscores the need for ongoing vigilance in patient selection and follow-up in the context of CXL. The rare development of corneal ectasia post-CXL highlights a gap in our understanding of corneal biomechanics and the potential limitations of current diagnostic tools. More research is needed to refine patient screening methods and optimize treatment protocols to ensure the efficacy and safety of this widely used procedure.
